# Altered Carnitine Metabolism in Ischemic and Non-Ischemic Cardiomyopathy: A Comparative Metabolomics Study Using LC–MS/MS

**DOI:** 10.3390/metabo15110685

**Published:** 2025-10-22

**Authors:** Yasemin Behram Kandemir, Ünal Güntekin, Veysel Tosun, İsmail Koyuncu, Özgür Yüksekdağ

**Affiliations:** 1 Department of Anatomy, Faculty of Medicine, Istanbul Aydın University, 34295 Istanbul, Turkey; 2 Department of Cardiology, Faculty of Medicine, Akdeniz University, 07070 Antalya, Turkey; unalguntekin@akdeniz.edu.tr; 3 Department of Cardiology, Mehmet Akif Inan Training and Research Hospital, University of Health Sciences, 63200 Sanliurfa, Turkey; veyseltosun@sbu.edu.tr; 4 Department of Medical Biochemistry, Faculty of Medicine, Harran University, 63300 Sanliurfa, Turkey; ikoyuncu@harran.edu.tr (İ.K.); ozguryuksekdag@harran.edu.tr (Ö.Y.)

**Keywords:** carnitine, acylcarnitine, ischemic cardiomyopathy, non-ischemic cardiomyopathy, metabolomics, LC–MS/MS

## Abstract

Background: Cardiomyopathy is a major cause of heart failure. Ischemic cardiomyopathy (IC) and non-ischemic cardiomyopathy (NIC) have distinct pathophysiological mechanisms. Carnitine plays a critical role in transporting long-chain fatty acids into mitochondria for β-oxidation. Disruptions in carnitine and acylcarnitine homeostasis have been implicated in cardiomyopathy; however, comparative profiling between IC and NIC remains limited. Methods: Serum samples were obtained from 40 IC patients, 40 NIC patients, and 40 age- and sex-matched controls. Free carnitine and 27 acylcarnitine species were quantified using LC–MS/MS. Multivariate analyses (PCA, PLS-DA), univariate statistics (ANOVA with Tukey’s HSD), and ROC curve analyses were performed to identify discriminatory metabolites and assess their diagnostic performance. Results: Compared with controls, IC patients exhibited reduced levels of short- and medium-chain acylcarnitines (C2, C4DC, C6, C8, C10, and C14), whereas NIC patients showed elevations in medium- and long-chain species (C6DC and C16). Heatmaps demonstrated clear group clustering. PCA and PLS-DA revealed partial separation, with C2, C6DC, and C16 emerging as the most influential metabolites (highest VIP scores). ROC analysis indicated modest diagnostic performance, with AUC values ranging from 0.623 to 0.635. Conclusions: IC and NIC are characterized by distinct alterations in serum carnitine profiles, reflecting differential metabolic remodeling. These findings may clarify disease mechanisms and highlight potential metabolic biomarkers or therapeutic targets. Acylcarnitine profiling could support differential diagnosis and personalized management in cardiomyopathy.

## 1. Introduction

Cardiomyopathy is clearly a major cause of morbidity and mortality worldwide and is classified into ischemic cardiomyopathy (IC), primarily caused by obstructive coronary artery disease, and non-ischemic cardiomyopathy (NIC), which encompasses a heterogeneous group of myocardial disorders including idiopathic, genetic, inflammatory, and metabolic etiologies [1,2]. Both conditions share common clinical features such as left ventricular systolic dysfunction and progressive heart failure; however, they differ significantly in their underlying pathophysiology, myocardial remodeling patterns, and metabolic adaptations [3,4,5].

Mitochondrial dysfunction is clearly a hallmark of both IC and NIC [1,2,3]. The myocardium is a high-energy–demanding tissue, with fatty acid oxidation (FAO) accounting for approximately 60–70% of ATP production under normal physiological conditions, supplemented by glucose, lactate, and ketone oxidation [6,7]. The efficient transport of long-chain fatty acids into the mitochondrial matrix is dependent on the carnitine shuttle, comprising carnitine palmitoyltransferase I (CPT I), carnitine-acylcarnitine translocase (CACT), and carnitine palmitoyltransferase II (CPT II) [8,9,10]. Free carnitine (C0) and its esterified form, acylcarnitines, are central intermediates in this process. Disruptions in carnitine metabolism impair FAO, resulting in the accumulation of toxic lipid intermediates, mitochondrial dysfunction, oxidative stress, and ultimately contractile failure [11,12,13,14]. However, most metabolomics studies have analyzed heart failure patients without distinguishing between IC and NIC, potentially masking etiology-specific metabolic fingerprints [4,5]. Recent studies have highlighted that metabolomics profiling can distinguish ischemic and non-ischemic heart failure etiologies, revealing distinct substrate utilization and energy remodeling patterns [15,16,17,18,19].

Acylcarnitines are increasingly recognized as metabolic biomarkers, not only for inherited metabolic disorders such as primary carnitine deficiency and fatty acid oxidation defects [20,21], but also for complex multifactorial conditions like heart failure, diabetes, and cardiovascular disease [22,23,24]. Altered serum acylcarnitine profiles have been reported in patients with reduced ejection fraction [25], with specific chain-length species reflecting distinct enzymatic defects or substrate utilization patterns [26]. In ischemic myocardium, hypoxia limits oxidative metabolism, potentially causing accumulation of incomplete β-oxidation products [27]. In contrast, NIC, often associated with chronic inflammatory or genetic alterations, may lead to alternative shifts in substrate metabolism and mitochondrial adaptation [28,29,30]. Identifying distinct acylcarnitine patterns between IC and NIC could provide novel diagnostic markers, improve disease classification, and reveal potential therapeutic targets [6,7].

Recent advances in targeted metabolomics, particularly through liquid chromatography–tandem mass spectrometry (LC–MS/MS), have enabled precise quantification of a wide range of acylcarnitines in biological samples [31,32,33]. This approach provides insight into mitochondrial function, identifies potential disease-specific metabolic signatures, and supports biomarker discovery for diagnosis, prognosis, and therapeutic monitoring [34,35,36]. In line with these advances, several recent studies using LC–MS/MS have successfully identified discriminative metabolic signatures in ischemic versus non-ischemic cardiomyopathy [15,16,19].

In this study, we aimed to (i) compare serum acylcarnitine profiles between IC, NIC, and healthy controls using LC–MS/MS, (ii) identify metabolites with significant discriminatory power, and (iii) explore their potential roles in cardiomyopathy pathophysiology. By integrating multivariate analysis with targeted biochemical quantification, we sought to reveal distinct metabolic remodeling patterns associated with different cardiomyopathy etiologies.

## 2. Materials and Methods

### 2.1. Ethical Approval

The study was approved by the Ethics Committee of Harran University Faculty of Medicine (Approval No: HRU/23.09.2024-152), Şanlıurfa, Turkey. All procedures were performed in accordance with the principles of the Declaration of Helsinki. Written informed consent was obtained from all participants.

### 2.2. Study Population

Eighty patients with cardiomyopathy (40 IC, 40 NIC) and 40 age- and sex-matched healthy controls were enrolled. Diagnosis was confirmed through clinical history, echocardiography, and angiographic data. Exclusion criteria included known metabolic disorders and carnitine supplementation. Baseline demographic and clinical characteristics of the study population are summarized in Table 1. These include age, sex, body mass index (BMI), ejection fraction (EF), New York Heart Association (NYHA) functional class, estimated glomerular filtration rate (eGFR), hypertension, diabetes mellitus, dyslipidemia, smoking status, and use of major cardiovascular medications (beta-blockers, ACEi/ARB/ARNI, MRA, SGLT2 inhibitors, and statins).

### 2.3. Sample Collection and Metabolite Analysis

After overnight fasting, venous blood samples were collected and serum was separated via centrifugation. Free carnitine (C0) and 27 acylcarnitine species were quantified in serum samples using liquid chromatography–tandem mass spectrometry (LC–MS/MS; Shimadzu North America, Columbia, MD, USA). Acylcarnitine quantification was performed using validated LC–MS/MS methods as described previously [37,38,39,40], enabling accurate detection of isomeric and odd-numbered acylcarnitines.

### 2.4. Statistical Analysis

Data were analyzed using SPSS version 24.0 (IBM Corp., Armonk, NY, USA) and MetaboAnalyst 5.0. Normality was assessed using the Shapiro–Wilk test. For normally distributed variables, one-way ANOVA with Tukey’s HSD was applied; for non-parametric data, the Kruskal–Wallis test with pairwise post hoc comparisons was used. To correct for multiple testing, false discovery rate (FDR) adjustment using the Benjamini–Hochberg method was applied. Multivariate analyses (PCA and PLS-DA) were performed to explore group separation. The robustness of the PLS-DA models was evaluated using Q^2^ statistics and 1000-permutation testing. Multiple logistic regression analyses were performed to assess discriminatory metabolites, adjusting for age, sex, EF, and medication use. Receiver operating characteristic (ROC) curves were generated to evaluate diagnostic performance. A *p*-value < 0.05 was considered statistically significant.

## 3. Results

### 3.1. Baseline Clinical Characteristics

A total of 120 participants were included: 40 with ischemic cardiomyopathy (IC), 40 with non-ischemic cardiomyopathy (NIC), and 40 age- and sex-matched healthy controls (HC). Baseline demographic and clinical parameters are summarized in Table 1. IC and NIC groups had similarly reduced left ventricular ejection fraction (EF 31.2 ± 7.6% vs. 33.8 ± 8.1%), whereas HC showed normal EF (62.4 ± 5.2%). NYHA functional class was predominantly III in both patient groups. Common comorbidities included hypertension, diabetes, and dyslipidemia, which were substantially more frequent in IC/NIC compared to controls. Most patients received contemporary guideline-directed therapy, including β-blockers, ACEi/ARB/ARNI, MRA, and SGLT2 inhibitors.

### 3.2. Overview of Serum Carnitine Profiles

Targeted LC–MS/MS analysis quantified free carnitine (C0) and 27 acylcarnitine species in all participants. Table 2 presents mean ± SD concentrations, ANOVA *p*-values, FDR-adjusted q-values, Tukey’s post hoc groupings, VIP scores, and adjusted odds ratios (OR) for key metabolites.

IC patients displayed a broad reduction in short-, medium-, and long-chain acylcarnitines, particularly C2 (acetylcarnitine), C3, C12, and C16, compared to controls (*p* < 0.05, FDR < 0.05). In contrast, NIC patients exhibited elevated C6DC (adipoylcarnitine) relative to both IC and controls (*p* < 0.05, FDR < 0.05).

Short-chain; C2 and C3 were significantly lower in IC than HC, suggesting impaired mitochondrial β-oxidation flux. Medium-chain; C12 was decreased in IC, whereas C6DC was increased in NIC, indicating differential dicarboxylic FA metabolism. Long-chain; C16 was lower in IC compared to HC, consistent with diminished fatty acid transport.

### 3.3. Distinction Between IC and NIC

**Figure 1:**
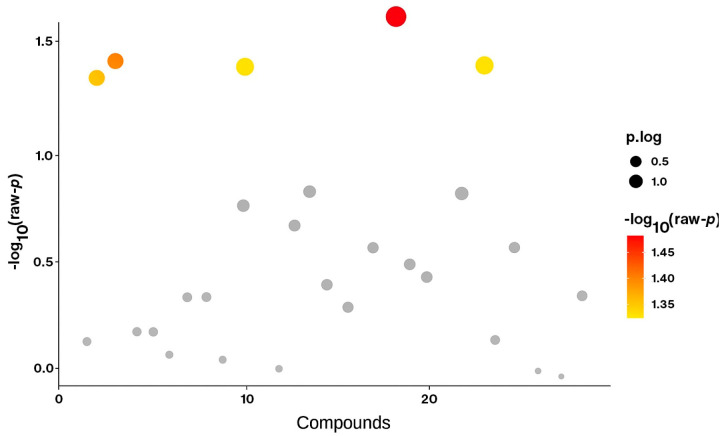
Volcano plot comparing metabolite levels between the ischemic cardiomyopathy (IC) and non-ischemic cardiomyopathy (NIC) groups using Student’s *t*-test. Each dot represents an individual acylcarnitine metabolite. The y-axis shows the −log10(*p*) values, where higher values indicate stronger statistical significance. Metabolites above the significance threshold (−log10(*p*) > 1.3, corresponding to *p* < 0.05) are highlighted in red/yellow, while non-significant metabolites are shown in grey. The size of the dots represents effect size (*p*.log).

The most striking difference between IC and NIC is the elevation of C6DC and C12 in NIC, suggesting that non-ischemic cardiomyopathy may involve specific alterations in dicarboxylic fatty acid metabolism not observed in ischemic disease (Figure 1). Overall, IC patients display a broader reduction in both short- and long-chain acylcarnitines, reflecting mitochondrial dysfunction due to ischemic injury.

### 3.4. Biological Implications

The reduction in C2, C3, C12, and C16 in IC highlights a consistent pattern of impaired fatty acid oxidation and diminished metabolic flexibility (Table 2 and Table 3). These metabolites, particularly C12 and C6DC, may serve as potential biomarkers for distinguishing ischemic from non-ischemic cardiomyopathy (Figure 1).

Compared with controls, ischemic cardiomyopathy patients exhibited significantly lower concentrations of C2, C3, C12, and C16, indicating impaired mitochondrial fatty acid metabolism. In contrast, non-ischemic patients showed higher C6DC levels, reflecting a distinct metabolic phenotype (Table 2 and Table 3). These findings support the role of acylcarnitine profiling in differentiating cardiomyopathy subtypes and identifying metabolic derangements.

### 3.5. Post Hoc Comparisons

The one-way ANOVA with subsequent Tukey’s post hoc analysis identified significant intergroup differences for several metabolites. Specifically, C12, C3, C2, and C16 exhibited significant reductions in the ischemic cardiomyopathy (IC) group compared with controls (HC), whereas C6DC was significantly elevated in the non-ischemic cardiomyopathy (NIC) group relative to controls (Table 3).

These results suggest that IC is characterized by a depletion of both short- and medium-chain acylcarnitines, reflecting impaired mitochondrial β-oxidation and limited acetyl-CoA availability for the tricarboxylic acid cycle. In contrast, the selective elevation of C6DC in NIC may indicate altered dicarboxylic fatty acid metabolism, potentially linked to peroxisomal oxidation or compensatory mitochondrial mechanisms.

Collectively, these findings reinforce the concept that distinct acylcarnitine signatures differentiate ischemic from non-ischemic cardiomyopathy. In particular, C12 and C2 appear to serve as sensitive markers of energy metabolic impairment in IC, while C6DC may represent a distinguishing feature of NIC. Such metabolite-specific alterations highlight the utility of targeted metabolomics in identifying pathophysiological differences among cardiomyopathy subtypes.

Metabolites with FDR-adjusted q < 0.05 were considered statistically significant. IC vs. C and NIC vs. C indicate which groups showed significant pairwise differences.

### 3.6. Multivariate Pattern Recognition

**Figure 2:**
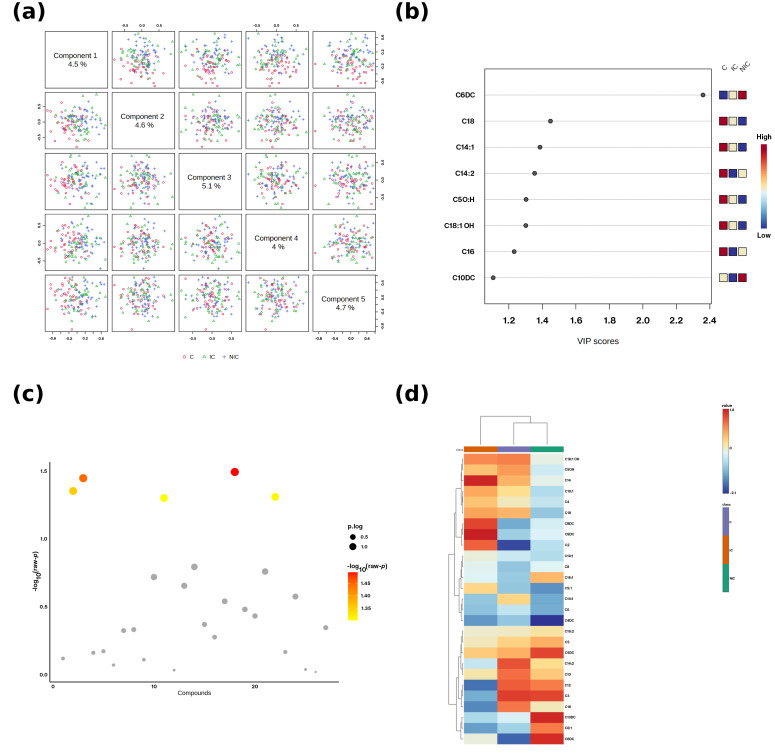
Multivariate and univariate summaries of acylcarnitines. (**a**) Scores scatterplots for Components 1-5 of the PLS-DA; each dot is on subject. Groups: C = Control, IC = Ischemic cardiomyopathy, NIC = Non-ischemic cardiomyopathy. (**b**) VIP scores for the top discriminant acylcarnitines. The small 3-cell pattern in the upperright corner encodes the direction of change per group (left-to-right: C, IC, NIC) using the adjacent color scale (blue = low, red = high relative to the cohort mean). (**c**) Bubble plot of-log_10_ (raw *p*) across compounds; point size reflects the p.log metric and point color maps to-log_10_(raw *p*). (**d**) Hierarchical-culustering heatmap of z-scored metabolite levels with sample and feature dendrograms; the color bar indicates relative abundance (blue = low, red = high).

Principal component analysis (PCA) showed partial separation between IC, NIC, and HC groups, while partial least squares-discriminant analysis (PLS-DA) demonstrated clearer class separation (R^2^ = 0.82, Q^2^ = 0.67, permutation *p* < 0.01). VIP analysis identified C2, C6DC, and C16 as the most discriminative variables (VIP > 1.2). A hierarchical clustering heatmap highlighted overall depletion of short/long-chain acylcarnitines in IC and selective C6DC enrichment in NIC (Figure 2a–d).

Principal component analysis (PCA) score plots (Figure 2a) revealed partial separation among ischemic cardiomyopathy (IC), non-ischemic cardiomyopathy (NIC), and control (HC) groups, indicating underlying differences in the acylcarnitine metabolic profile. Variable importance in projection (VIP) analysis (Figure 2b) identified several metabolites, such as C6DC, C3, C14:1, C14:2, C5OH, C18:1OH, C16, and C10DC, as key contributors to group discrimination, with VIP scores > 1.2. The volcano plot (Figure 2c) highlighted statistically significant metabolites, with increased or decreased levels across groups, and the hierarchical clustering heatmap (Figure 2d) demonstrated distinct clustering patterns, where IC and NIC groups showed differential acylcarnitine abundance compared to controls.

### 3.7. Differential Metabolite Significance

Volcano analysis (Figure 2c) confirmed that C2, C3, C12, and C16 were significantly downregulated in IC, while C6DC was significantly upregulated in NIC. These results indicate that IC is characterized by global energy metabolism impairment, whereas NIC maintains partial FAO activity but with a shift toward dicarboxylic fatty acid pathways.

### 3.8. Diagnostic Potential of Key Metabolites

**Figure 3:**
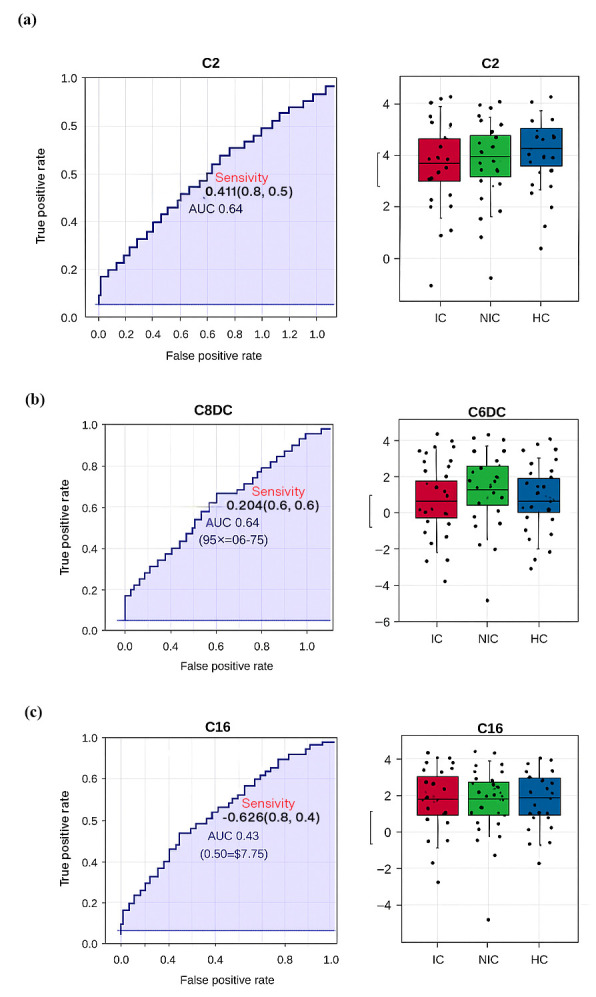
(**a**) ROC (Receiver Operating Characteristic) analysis for the C2 (Acetylcarnitine) metabolite, with an AUC (Area Under the Curve) value of 0.633, indicating limited discriminatory power better than random guessing but below the threshold for a strong biomarker. The boxplot shows a moderate separation between groups, though overlaps remain. (**b**) ROC analysis for the C6DC (Adipoylcarnitine) metabolite yielded an AUC of 0.635, reflecting performance above random but with moderate diagnostic value. Boxplot analysis reveals that NIC groups had the highest levels, while control groups had the lowest. (**c**) ROC analysis for the C16 (Palmitoylcarnitine) metabolite produced an AUC of 0.623, indicating limited predictive power. Boxplots show the highest levels in control groups, intermediate levels in NIC groups, and lower distributions in IC groups.

Figure 3a (ROC for C2) yielded an AUC of 0.633, indicating modest discrimination, with lower C2 in IC as the main driver. Figure 3b (ROC for C6DC) achieved an AUC of 0.635, with the highest values in NIC, intermediate in IC, lowest in controls. Figure 3c (ROC for C16) showed an AUC of 0.623, with the highest levels in controls, moderate in NIC, lowest in IC. Although AUC values suggest limited standalone biomarker potential, these metabolites may improve discrimination when combined in a panel.

Receiver operating characteristic (ROC) curves (Figure 2) were generated for key metabolites to assess their diagnostic potential. C2 showed an area under the curve (AUC) of 0.633 for discriminating IC from controls (Figure 2a), C6DC had an AUC of 0.634 for distinguishing NIC from controls (Figure 2b), and C16 had an AUC of 0.623 for differentiating IC from controls (Figure 2c). Although individual metabolites demonstrated moderate discriminative power, combining multiple acylcarnitines may enhance classification accuracy.

Overall, IC was characterized by widespread reductions in short-, medium-, and long-chain acylcarnitines, consistent with mitochondrial β-oxidation impairment and reduced energy production. NIC displayed a more heterogeneous metabolic pattern, with partial preservation of fatty acid oxidation and evidence of increased branched-chain amino acid catabolism. These data suggest that C2, C16, C14:2, C5OH, and C6DC are potential metabolic markers for differentiating cardiomyopathy subtypes.

## 4. Discussion

In this targeted metabolomics study, we demonstrated distinct alterations in serum acylcarnitine profiles between ischemic cardiomyopathy (IC) and non-ischemic cardiomyopathy (NIC). Although both conditions share progressive myocardial dysfunction, our findings suggest etiology-specific metabolic remodeling that is detectable in peripheral blood and likely reflects underlying differences in mitochondrial function, substrate utilization, and adaptive responses.

In IC patients, the marked reduction of short-chain acylcarnitines, especially C2 (acetylcarnitine) and C3, indicates impaired mitochondrial β-oxidation and reduced acetyl-CoA availability for the tricarboxylic acid cycle. Ischemic hypoxia is known to inhibit long-chain acyl-CoA dehydrogenase and carnitine palmitoyltransferase (CPT) activity, thereby limiting fatty acid oxidation (FAO) capacity [1,2,3,4,5,32,33,34]. C2 is a key metabolite that buffers acetyl-CoA and supports metabolic flexibility; its depletion may exacerbate energetic failure in ischemic myocardium.

Conversely, NIC, which is frequently associated with dilated or inflammatory cardiomyopathy, showed selective accumulation of medium- and long-chain species, notably C6DC and C16. This may indicate incomplete β-oxidation due to enzymatic bottlenecks at the level of acyl-CoA dehydrogenases or peroxisomal overload [6,7,35,36,37]. Long-chain acylcarnitines can impair mitochondrial membrane potential and promote reactive oxygen species production, potentially contributing to the higher arrhythmic risk reported in NIC [8,9,10,38,39].

Our findings align with recent metabolomics studies that reported similar chain-length-specific patterns in heart failure subtypes [41,42,43,44,45]. However, studies of end-stage heart failure often report global elevations across all chain lengths [14,15,16,17,18,19,20,21], suggesting that metabolic specificity is lost in advanced disease. Our cohort likely represents earlier to mid-stage disease, where residual etiology-specific patterns are still present.

Mechanistically, our results may be explained by: (i) Ischemic hypoxia model; oxygen deficiency suppresses FAO, depleting downstream short-chain metabolites; (ii) Enzymatic dysfunction model; genetic or inflammatory insults impair specific β-oxidation steps, leading to selective medium-/long-chain accumulation; (iii) Carnitine transport dysregulation; impaired CPT1/2 or carnitine-acylcarnitine translocase (CACT) function causes accumulation of toxic intermediates [22,23,24].

The diagnostic performance of individual metabolites was modest (AUC 0.62–0.64). Although not suitable as stand-alone biomarkers, combining C2, C16, and C6DC into multi-marker panels alongside clinical and imaging parameters could enhance diagnostic discrimination [25,26,27,28]. Machine learning approaches have shown improved classification of cardiac phenotypes using metabolomics [29,30]. Longitudinal monitoring may also capture disease progression and treatment response [31].

Therapeutically, restoring depleted carnitine pools in IC (e.g., L-carnitine or acetyl-L-carnitine supplementation) and promoting complete β-oxidation in NIC (e.g., PPAR agonists or trimetazidine) are potential strategies [32,33,34,35,36,39]. Stratifying patients based on metabolomics fingerprints may improve response to such targeted therapies.

This study has several limitations. The sample size is relatively small, which limits the generalizability of our findings, and the design is cross-sectional, precluding causal inference. Dietary intake and physical activity were not standardized and may have affected carnitine metabolism. Furthermore, our findings are exploratory and hypothesis-generating, and require validation in larger, multicenter cohorts to assess their reproducibility and clinical utility.

## 5. Conclusions

This study reinforces the concept that cardiomyopathy subtypes have distinct metabolic fingerprints. By identifying specific acylcarnitine patterns associated with IC and NIC, our study highlights the potential of metabolomics to inform diagnosis, prognosis, and targeted therapy. Ultimately, incorporating metabolic biomarkers into the routine evaluation of cardiomyopathy may refine patient stratification and management.

## Figures and Tables

**Table 1:** Baseline Characteristics of the Study Population.

Characteristics	IC (n = 40)	NIC (n = 40)	HC (n = 40)
Age (years)	62.8 ± 9.4	60.7 ± 10.1	59.3 ± 8.7
Male sex, n (%)	30 (75.0%)	28 (70.0%)	26 (65.0%)
BMI (kg/m^2^)	28.1 ± 3.9	27.5 ± 4.1	26.7 ± 3.8
EF (%)	31.2 ± 7.6	33.8 ± 8.1	62.4 ± 5.2
NYHA class (I/II/III/IV)	0/6/22/12	1/10/21/8	—
eGFR (mL/min/1.73 m^2^)	72.5 ± 18.9	75.4 ± 17.3	88.6 ± 15.2
Hypertension, n (%)	25 (62.5%)	23 (57.5%)	9 (22.5%)
Diabetes mellitus, n (%)	18 (45.0%)	15 (37.5%)	4 (10.0%)
Dyslipidemia, n (%)	22 (55.0%)	21 (52.5%)	7 (17.5%)
Smoking, n (%)	17 (42.5%)	16 (40.0%)	10 (25.0%)
BB, n (%)	38 (95.0%)	36 (90.0%)	—
ACEi/ARB/ARNI, n (%)	35 (87.5%)	33 (82.5%)	—
MRA, n (%)	29 (72.5%)	27 (67.5%)	—
SGLT2i, n (%)	18 (45.0%)	16 (40.0%)	—
Statin, n (%)	30 (75.0%)	28 (70.0%)	—
Characteristics	IC (n = 40)	NIC (n = 40)	HC (n = 40)
Age (years)	62.8 ± 9.4	60.7 ± 10.1	59.3 ± 8.7
Male sex, n (%)	30 (75.0%)	28 (70.0%)	26 (65.0%)
BMI (kg/m^2^)	28.1 ± 3.9	27.5 ± 4.1	26.7 ± 3.8
EF (%)	31.2 ± 7.6	33.8 ± 8.1	62.4 ± 5.2
NYHA class (I/II/III/IV)	0/6/22/12	1/10/21/8	—
eGFR (mL/min/1.73 m^2^)	72.5 ± 18.9	75.4 ± 17.3	88.6 ± 15.2
Hypertension, n (%)	25 (62.5%)	23 (57.5%)	9 (22.5%)
Diabetes mellitus, n (%)	18 (45.0%)	15 (37.5%)	4 (10.0%)
Dyslipidemia, n (%)	22 (55.0%)	21 (52.5%)	7 (17.5%)
Smoking, n (%)	17 (42.5%)	16 (40.0%)	10 (25.0%)
BB, n (%)	38 (95.0%)	36 (90.0%)	—
ACEi/ARB/ARNI, n (%)	35 (87.5%)	33 (82.5%)	—

Data are presented as the mean ± SD or n (%). IC: ischemic cardiomyopathy; NIC: non-ischemic cardiomyopathy; HC: healthy controls; BMI: body mass index; EF: ejection fraction; NYHA: New York Heart Association; eGFR: estimated glomerular filtration rate. BB: beta-blockers; ACEi: angiotensin-converting enzyme inhibitors; ARB: angiotensin II receptor blockers; ARNI: angiotensin receptor–neprilysin inhibitors; MRA: mineralocorticoid receptor antagonists; SGLT2i: sodium–glucose cotransporter-2 inhibitors.

**Table 2:** Serum concentrations (mean ± SD) of free carnitine (C0) and 27 acylcarnitines in ischemic cardiomyopathy (IC), non-ischemic cardiomyopathy (NIC), and control (HC) groups. Values were analyzed using one-way ANOVA with Tukey’s post hoc test.

Carnitine	IC (Mean ± SD)	NIC (Mean ± SD)	HC (Mean ± SD)	*p*	FDR (q)	Post Hoc	VIP	Adj.OR (*p*)	
C0	207.61 ± 75.21 ^a^^b^	217.46 ± 112.53 ^a^	204.99 ± 85.07 ^a^^b^	ns	—	—	1.05	—	
C2	4.23 ± 4.34 ^a^	5.41 ± 4.54 ^a^	9.05 ± 8.04 ^b^	<0.05	0.021	IC<C	1.31	2.1 (0.03)	
C3	0.58 ± 0.44 ^a^	0.84 ± 0.75 ^a^^b^	0.98 ± 0.68 ^b^	<0.05	0.027	IC<C	1.25	1.6 (0.06)	
C4	0.66 ± 0.49	0.89 ± 0.88	0.76 ± 0.52	ns	—	—	1.00	—	
C4DC	0.04 ± 0.02	0.05 ± 0.03	0.06 ± 0.03	ns	—	—	1.02	—	
C5	0.55 ± 0.31	0.59 ± 0.34	0.53 ± 0.24	ns	—	—	1.01	—	
C5:1	0.05 ± 0.03	0.06 ± 0.06	0.06 ± 0.03	ns	—	—	0.95	—	
C5OH	0.08 ± 0.04	0.11 ± 0.06	0.08 ± 0.04	ns	—	—	1.12	—	
C5DC	0.27 ± 0.19	0.43 ± 0.50	0.58 ± 0.36	ns	—	—	1.08	—	
C6	0.14 ± 0.12 ^a^	0.22 ± 0.14 ^b^	0.23 ± 0.17 ^b^	<0.05	0.041	NIC>C	1.18	—	
C6DC	0.07 ± 0.05 ^a^	0.09 ± 0.08 ^b^	0.09 ± 0.05 ^b^	<0.05	0.018	NIC>C	1.53	2.4 (0.02)	
C8	0.33 ± 0.29	0.45 ± 0.30	0.59 ± 0.32	ns	—	—	1.09	—	
C8:1	0.32 ± 0.33	0.52 ± 0.47	0.55 ± 0.57	ns	—	—	1.11	—	
C8DC	0.02 ± 0.02	0.03 ± 0.03	0.04 ± 0.03	ns	—	—	0.93	—	
C10	0.49 ± 0.46	0.60 ± 0.38	0.94 ± 0.56	ns	—	—	1.15	—	
C10:1	0.26 ± 0.19	0.40 ± 0.26	0.67 ± 0.43	ns	—	—	1.20	—	
C10DC	0.03 ± 0.01	0.03 ± 0.01	0.03 ± 0.01	ns	—	—	0.97	—	
C12	0.13 ± 0.11 ^a^	0.14 ± 0.08 ^a^^b^	0.20 ± 0.11 ^b^	<0.05	0.033	IC<C	1.42	1.8 (0.04)	
C14	0.08 ± 0.06	0.09 ± 0.05	0.11 ± 0.07	ns	—	—	1.05	—	
C14:1	0.05 ± 0.04	0.08 ± 0.06	0.14 ± 0.08	ns	—	—	1.28	—	
C14:2	0.01 ± 0.01	0.03 ± 0.02	0.04 ± 0.02	ns	—	—	1.31	—	
C16	0.24 ± 0.11 ^a^	0.27 ± 0.11 ^a^^b^	0.33 ± 0.19 ^b^	<0.05	0.029	IC<C	1.35	1.9 (0.04)	
C16:1	0.03 ± 0.02	0.05 ± 0.03	0.09 ± 0.05	ns	—	—	1.26	—	
C18	0.10 ± 0.05	0.12 ± 0.07	0.14 ± 0.08	ns	—	—	1.04	—	
C18:1	0.08 ± 0.08	0.16 ± 0.12	0.21 ± 0.13	ns	—	—	1.19	—	
C18:2	0.04 ± 0.03	0.08 ± 0.05	0.10 ± 0.06	ns	—	—	1.16	—	
C18:1OH	0.03 ± 0.02	0.02 ± 0.01	0.04 ± 0.02	ns	—	—	1.22	—	

Different superscript letters (^a^^,^^b^^,^^a^^b^) indicate group differences. Groups sharing the same letter are not significantly different, whereas groups with different letters are significantly different (Tukey’s post hoc, *p* < 0.05, FDR-adjusted). For example, IC (^a^) vs. C (^b^) are significantly different, whereas NIC (^a^^b^) is not significantly different from either IC or HC.

**Table 3:** Post hoc ANOVA Results for Differentially Expressed Acylcarnitines.

Carnitin	F-Value	*p* -Value	−log10(*p*)	FDR	Post Hoc Tests
C12	3.5426	<0.005 ^a^	1.4938	0.26948	IC-C
C3	3.4308	<0.005 ^a^	1.4479	0.26948	IC-C
C2	3.2019	<0.005 ^a^	1.3538	0.26948	IC-C
C16	3.0958	<0.005 ^a^	1.3101	0.26948	IC-C
C6DC	3.0758	<0.005 ^a^	1.3019	0.26948	NIC-C

Post hoc pairwise comparisons of selected serum acylcarnitines among ischemic cardiomyopathy (IC), non-ischemic cardiomyopathy (NIC), and healthy control (HC) groups were performed using one-way ANOVA followed by Tukey’s HSD test. ^a^, *p* < 0.05. “^a^” Statistical comparisons were performed using a post hoc Tukey’s test.

## Data Availability

The datasets generated and analyzed during the current study are available from the corresponding author upon reasonable request. The data are not publicly available due to privacy and ethical restrictions.

## Abbreviations

C0: free carnitine, C2: acetylcarnitine, C3: propionylcarnitine, C3-DC: malonylcarnitine, C4: butyrylcarnitine, C4-OH: hydroxybutyrylcarnitine, C4-DC: succinylcarnitine, C5: isovalerylcarnitine, C5-OH: hydroxyisovalerylcarnitine, C5-DC: glutarylcarnitine, C6: hexanoylcarnitine; C6-DC: adipoylcarnitine, C8: octanoylcarnitine, C8:1: octenoylcarnitine, C10: decanoylcarnitine, C10:1: decenoylcarnitine, C10:2: decadienoylcarnitine, C12: dodecanoylcarnitine, C12:1: dodecenoylcarnitine, C14: tetradecanoylcarnitine, C14:1: tetradecenoylcarnitine, C14:2: tetradecadienoylcarnitine, C16: palmitoylcarnitine, C16-OH: hydroxypalmitoylcarnitine, C16:1: palmitoleylcarnitine, C18: stearoylcarnitine, C18:1: oleoylcarnitine, C18:2: linoleoylcarnitine, and C18:1OH: hydroxy_oleylcarnitine.
